# Can a genetic signature for metastatic head and neck squamous cell carcinoma be characterised by comparative genomic hybridisation?

**DOI:** 10.1038/sj.bjc.6601756

**Published:** 2004-04-13

**Authors:** H S Patmore, N E Ashman James, L Cawkwell, A MacDonald, N D Stafford, J Greenman

**Affiliations:** 1 Postgraduate Medical Institute of the University of Hull, Hull and York Medical School, University of Hull, Cottingham Road, Hull HU16 5JQ, UK; 2 Department of Histopathology, Hull Royal Infirmary, Anlaby Road, Hull HU3 2JZ, UK

**Keywords:** comparative genomic hybridisation, head and neck cancer, metastasis

## Abstract

Survival from head and neck squamous cell carcinoma (HNSCC) has remained static for the last 20 years. The development of lymph node metastasis (LNM) significantly reduces the 5-year survival rate, thus the ability to identify tumours with the potential to metastasise would allow more aggressive treatment regimes to be directed at these patients regardless of negative clinical and radiological findings at the time of presentation. Comparative genomic hybridisation (CGH) can identify chromosomal aberrations that may lead to metastasis. DNA from 23-paired specimens of primary tumour (PT) and LNM were analysed. Nonrandom copy number changes were identified in all paired samples. Similar numbers of aberrations were identified on PT and LNM samples. The most common aberrations were 3q (90%), 8q (65%), 1q (50%), 5p (43%), 2q (41%) and 11q (41%) and deletions 3p (57%), 1p (54%), 4p (48%), 13q (48%), 11q (41%) and 10q (37%). A number of differences were also detected. No aberration was found to be preferentially associated with the LNM, although gains on 6q (48 *vs* 22%) and 22q (26 *vs* 9%) were found at higher frequencies. Clonality studies demonstrated that LNM develop from the dominant population of cells in the PT. These results were compared with two similar publications. No combination of chromosomal aberrations, as detected by CGH, was associated with metastatic progression in HNSCC.

Head and neck squamous cell cancer (HNSCC) is a major health problem worldwide accounting for significant morbidity and mortality. The survival rate over the last 20 years has not improved appreciably despite a number of new and refined treatment modalities. Although many different prognostic indicators have been studied, the occurrence of nodal metastasis at presentation remains the single most important factor, decreasing the 5-year survival rate from approximately 75 to 29% (Ono, 1986). The identification of tumours with metastatic potential prior to the occurrence of detectable nodal spread would allow more aggressive primary treatment regimes to be instigated and directed at regional as well as local control.

Genetic studies have begun to characterise the chromosomal abnormalities involved in HNSCC. However, relatively little is known about the genetic aberrations associated with the metastatic event. Comparative genomic hybridisation (CGH) is a powerful technique that screens the entire genome for regions of DNA sequence copy number alterations. Comparative genomic hybridisation has previously been applied in several HNSCC studies and has demonstrated a nonrandom pattern of genomic aberrations commonly showing deletions of material from 3p, 4q, 5q, 9p, 18q and gains involving 3q, 5p, 7p, 8q, 11q, 17q and 20q (Struski *et al*, 2002). Although a number of tumour progression models have been constructed, based on the frequency of these aberrations (Bockmuhl *et al*, 2002; Huang *et al*, 2002), there has been no clear pattern of mutations that lead to metastasis.

An additional level of complexity is found in HNSCC due to the coexistence of karyotypically unrelated clones (Veltman *et al*, 1998; Jin *et al*, 2002). The presence of multiple clones with differing phenotypic characteristics within the primary can make identification and analysis of the metastatically competent cells very difficult.

Only a few studies have looked at the direct comparison between complete karyotypes of HNSCC primary tumour (PT) and the related lymph node metastases (LNM) (Kujawski *et al*, 1999; Bockmuhl *et al*, 2002). Kujawski *et al* (1999) compared data from CGH analysis of 19 paired laryngeal SCC PT and LNM. Out of 17 analysed pairs, the most common copy number changes on both primary tumour and metastasis were found on 3p, 3q, 5p, 9 and 13. Losses on chromosomes 13, 8p and 9q were found more frequently on the LNM than the PT. Welkoborsky *et al* (2000) analysed 20 patient–10 node-negative and 10 node-positive (both primary and lymph node samples) oropharyngeal and hypopharyngeal squamous cell carcinoma (SCC). Over-representations, as defined by these authors, of 21q and 22q were found in both PT and LNM. Gains of 1q, 8q, 11q, 18q and 19p were more frequent in the LNM, whereas a gain on 11p and a loss on 17p were only found in the LNM. Unfortunately, the results from Welkoborsky *et al* (2000) could not be used for comparative purposes, as the raw data were not shown in the publication. Bockmuhl *et al* (2002) investigated 54 patients: 34 matched HNSCC pairs and 20 nonmetastasising HNSCC samples. The clonal relationship between the PT and LNM, that is, evidence of the LNM originating from the PT was evaluated using three mathematical models, revealing concordance in 64–68% of the matched pairs. Of the paired samples, gains on 11q13, 7q11.2, 1q21–q22 and deletions on 8p, 11p14-qter, 10p12, 10q and 14 were associated with metastasis. Owing to the relatively low numbers of matched HNSCC (PT and LNM) that have been analysed, we describe the second largest cohort of CGH data for primary HNSCC and their corresponding lymph nodes. The possibility of identifying potential genetic aberrations that lead to metastasis is discussed after combining our data with the results of the two previous studies.

## MATERIALS AND METHODS

### Tissue samples

Tissue samples were collected from 23 patients (17 male and six female) undergoing surgery for HNSCC at the Department of Otolaryngology and Head and Neck Surgery, Hull Royal Infirmary between 1997 and 2002. All biopsies were collected from patients undergoing composite resections for oral cavity and oropharyngeal tumours, or total laryngectomy with or without partial pharyngectomy for tumours of the laryngopharynx. In each case, the LNM and primary biopsy were taken at the same time and no second primary was evident in any of the cases. There was neither a history of previous malignancy nor had any of the patients received treatment prior to surgical resection. A consultant pathologist (A.M) confirmed all samples to be SSC and all tumours were staged according to UICC guidelines. The Hull and East Yorkshire Research Ethics Committee approved the study and informed, written consent was obtained from all patients. Samples were immediately snap frozen in liquid nitrogen at the time of resection and stored at −80°C. The clinicopathological details of all patients are summarised in Table 1

**Table 1:** Patients clinicopathological details

**Patient no.**	**Age (years)**	**Tumour site**	**T stage**	**N stage**	**Differentiation**
1	58	Hypopharynx	4	2	Poor
2	53	Hypopharynx	4	3	Poor
3	55	Larynx	4	2c	Poor
4	55	Hypopharynx	1	2	Poor
5	54	Oropharynx	4	1	Moderate
6	65	Larynx	3	1	Moderate
7	70	Larynx	2	1	Well
8	50	Hypopharynx	4	2b	Poor
9	67	Hypopharynx	4	2	Poor
10	52	Larynx	4	2c	Poor
11	71	Larynx	4	1	Moderate
12	57	Oropharynx	2	2	Poor
13	65	Oropharynx	4	2b	Moderate
14	67	Hypopharynx	3	2a	Poor
15	55	Hypopharynx	3	2a	Moderate
16	61	Larynx	3	2b	Poor
17	48	Larynx	3	1	Mod
18	65	Hypopharynx	2	2	Poor
19	44	Larynx	2	2	Moderate
20	72	Hypopharynx	4	1	Poor
21	56	Larynx	4	2	Poor
22	76	Larynx	4	2	Moderate
23	61	Larynx	4	2c	Moderate

T stage=tumour stage; N stage=nodal stage.

. For each sample, a 10 μm tissue section was cut adjacent to the extracted section. These were stained with haematoxylin and eosin to assess normal to tumour cell ratio; greater than 70% tumour cell content was needed for CGH analysis.

### Comparative genomic hybridisation

DNA was extracted from 20 serial 20 *μ*m cryostat sections, or using the whole biopsy by proteinase K digestion followed by phenol–chloroform extraction. DNA quality and purity were assessed by electrophoresis and spectrophotometry at 260 nm and CGH performed essentially as described previously (Stafford *et al*, 1999). All CGH reagents were obtained from Vysis Ltd (Maidenhead, UK). All experiments were performed in combination with both positive (DNA with known aberrations) and negative (normal: normal hybridisation) control experiments. Sex mismatching of test and reference DNA precluded the analysis of the sex chromosomes. Deletions and gains of DNA were identified whenever the CGH ratio profile exceeded thresholds established through normal: normal hybridisations (0.85 and 1.15, respectively).

### Analysis

The presence of each aberration was scored independently, that is, gains and deletions were considered separately. Multiple gains or deletions on the same arm were interpreted depending on the site, size and amplification involved according to the guidelines in Zitzelsberger *et al*, (1997) and Jeuken *et al*, (2002).

### Clonal relationship

If the LNM originated from the dominant cell population within the PT, similarities between the copy number changes and their location would be expected. Based on this expectation, Alejandro Schaffer developed a probabilistic model to quantify the association between two matched tumour samples (Kuukasjarvi *et al*, 1997).

The occurrence of an aberration was weighted depending on its frequency in the entire population. Aberrations common to both paired samples could then be used to evaluate the clonality. ‘This model uses only data from shared abnormalities and assumes that the paired specimens must be both losses or both gains, and that the breakpoint along the chromosome arm is the same’ (Kuukasjarvi *et al*, 1997).

### Statistical analysis

If there is a clonal relationship between the two groups of specimens, the occurrence of an aberration in the primary would alter the expectation of its presence in the LNM, thus McNemar's test was applied to evaluate the significance of the individual aberrations. *χ*^2^ or Fisher's exact test, as appropriate, was also used so that the comparison with other publications could be performed.

## RESULTS

Comparative genomic hybridisation analysis of our 46 samples revealed a mean of 18 (range 5–36) and 19 (range 7–43) aberrations in the PT and LNM, respectively. From these data, a mean of 11 aberrations were found common to both samples. The follow-up and survival data from these patients have been discussed previously (Ashman *et al*, 2003).

The most frequent aberration was found to be a gain on 3q, observed in 21 out of the 23 patients (91%), more precisely the loci 3q25–q27 in 20 of the 23 of the PT (87%), and 21 of the 23 LNM (91%). Table 2

**Table 2:** Frequency of chromosomal aberrations found in greater than 30% of the specimens analysed

		**Frequency (*n*=23)**		**Bockmuhl *et al* (2002) (*n*=34)**		**Kujawski *et al* (1999) (*n*=17)**	
**Location**	**Gain/deletion**	**% PT**	**% LNM**	**% PT**	**% LNM**	**% PT**	**% LNM**
3q26–27	Gain	78	87	80	85	59	71
3p25-pter	Deletion	52	43	83	80	53	41
5q34-qter	Deletion	52	39	50	70	6	12
1p34.2-pter	Deletion	43	39	65	27	0	0
5p15.1-pter	Gain	43	22	50	50	35	29
11q13.3–13.5	Gain	39	26	65	80	47	53
11q23.3	Deletion	39	30	65	58	21	32
12p12.3–13.1	Gain	39	26	40	50	29	12
2q31	Gain	39	35	60	48	6	6
3q24	Gain	39	65	80	60	41	35
8q21.3	Gain	39	30	50	50	0	18
8q23	Gain	39	57	60	80	6	18
18p11.31-pter	Gain	35	26	23	20	0	5
3p14.1–3	Deletion	35	39	85	87	71	59
6q12	Gain	35	39	55	35	0	6
10q26.1-qter	Deletion	30	35	45	38	5	5
13q31	Deletion	30	43	85	67	47	71
4p16	Deletion	30	43	55	60	29	35
8p22	Deletion	30	30	55	60	6	24
1q31	Gain	26	30	40	43	24	24
7q31.1–3	Gain	26	30	44	38	5	16
12q22	Gain	22	30	45	37	6	0
1q21.2	Gain	22	30	75	65	24	24
19p13.3-pter	Deletion	17	30	15	63	0	0
4q26	Gain	17	30	77	27	0	0
4q34	Deletion	17	35	70	55	18	24
6q22	Gain	17	35	50	50	0	0
5q13	Deletion	13	35	69	75	32	37
9p24	Deletion	13	30	56	69	42	47
13q22	Gain	9	30	85	70	0	5

It should be noted that the percentage shown in Bockmühl's series is the maximum percentage found at any particular location read from the histogram provided on http://amba.charite.de/cgh. PT=primary tumour; LNM=lymph node metastasis.

summarises the frequent aberrations, greater than 30% found in either or both of the matched specimens. For comparison, the data from Bockmühl's and Kujawski's series are included in Table 2.

A mean of seven and eight aberrations were found to be unique to either PT or LNM, respectively. The most common unique aberration found throughout the metastatic samples was a gain on 6q present in nine of 23 (39%) lymph nodes. This gain was found in four other lymph nodes, but also in their relevant PT; two PT had unique aberrations on 6q. The gains on 6q were further located to two independent loci, 6q13 and 6q22.3. A gain on 22q was unique to the LNM at a slightly lower frequency–six of 23 (26%). One matched pair and one PT independent of its lymph node expressed this aberration as well. Other frequent findings unique to the lymph nodes were gains on 1q, 7q, 13q and deletions on 1p. McNemar's and Fisher's exact tests did not find any significant differences between the expression of specific locations in either PT and LNM genotypes.

The chromosomal locations of all of the aberrations are shown in Tables 3

**Table 3:** Chromosomal locations of DNA gain in individual patients

**ID no.**	**Both samples, common gains**	**PT, unique gains**	**Total no. gains**	**LNM, unique gains**	**Total no. gains**
1	3q, 11q13	1q25-qter, 6p22, 8q21–23,12p	6	1p33–35, 16, 17, 18p, 19, 20q, 22	9
2	3q	7qcen-q32, 11q13, 18q11.2	4	1q21–32, 22q	3
3	1q21–31, 3q22-qter, 5p, 8q, 11p13–q22		5	4q13.1–13.3, 6qcen-23, 12q21.1–21.3, 22q	9
4	1q25–31		1	3q, 6q21, 8q22–24, 18q	5
5	1p22, 3q24-qter, 7q21–31.2, 11q13	6p22-pter	5	5p, 6qcen-15, 13q21–22	7
6	3q, 8, 12p11.2–q 23	5p, 7p15–q32, 13q14, 14qcen-q22, 16p11.2–12, 18p	9	1p22, 2q22–32, 4cen-q31, 5qcen-23, 6qcen-23, 9p21, 11p13, 13q21–31	11
7		7q11.2, 18q11.2	2	2p16, 2q22–q32, 3q25–q26, 4p14–q31, 5q21–q23, 6qcen-q23,12q21,13q2-q31	8
8	1qcen-q32, 3q, 5p, 8q, 11q22,	2q24.3, 9q, 12q21.3, 14q22–23	9	13q21	6
9	5pcen-p14, 7cen, 12p	3q25.2–q25.3	4	2q32.3, 6qcent-q12, 6q21–q22.3, 11q21	7
10	2q22–31, 3q24–26, 4qcen-q31, 6qcen-23, 8q, 12q21	1p31–22, 5p, 5q15–23, 11p14, 11q21–22, 13q, 18q12	13		6
11	2q22–q32, 3cen-q13, 3q24–q27, 8q, 12q21, 13q21	2p13–p16, 7q11.2, 12p	9	1p22, 4q, 5q21–22, 6qcen-q21, 9p21	11
12	3q	6qcen-q21	2	1p32–p35, 12p13, 16p, 17q12, 19q, 20q, 22	8
13	1pcen-p22, 2q22–32, 3q24-qter, 4cen-q25, 6qcen-q22, 12q15–21	8q21–23, 9q, 11p13, 14q13	10	13q21, 17q23-qter	8
14	3q24-qter, 5p, 6p22-pter, 8q, 13q31-qter,18	1q32-qter, 6qcen-q16	8	5q34-qter, 7q11.2, 15cen-q14, 20p	10
15	3q24–q28, 4qcen-q21.1, 5p, 8q23, 13q21.2–q21.3, 18p11.31-pter, 22q12.2–q13.2	2p, 4p16-pter, 7p21, 12p, 16p13.2-pter, 17p13-pter	13	20q13.2	8
16	1q, 2p13–p21, 2q24–q32, 3q, 6qcen-q22, 7cen-q33, 8q, 11q13, 14q, 18p	17cen-q21, 20cen-q12	12	9q31	11
17	2q22–q32, 3q, 4cen-q31, 8q, 11p14–q13	5p, 12p13-pter, 19q	8	1q31, 6q22–q24, 7q31.1–31.3, 18p	9
18	1q21.1-qter, 2q21.3–32.1, 3q21-qter, 5p15.1-pter, 8q22.2–24.2, 15q15–26.1, 18p11.31-pter	2p14–16, 6p11.2-pter, 7pcent-15.3, 7q33-qter, 7q21.1–21.3, 12p, 16q12.1–23	14	7p15.3–p21, 7q31.1–31.3, 11pcent-13, 14qcent–13	11
19	2pcent-11.2, 2p13-pter, 3q23-qter, 5p, 9p21–p22, 9q22.2–q33, 14q24.1–q32, 17p, 17qcent-q23, 19pcent-p13.1	2q24.3–q32.1, 6p11.2–p21.1, 7q21.3–q31.1, 7q36, 11qcent-q13.3, 12pcent-12.1, 22qcent-13.2	17	1q21–23, 7pcent-p14, 10p11.1–p11.2, 10p15, 12qcent-q21.1, 14qcent-q21, 18p	17
20	2q24.1–q32, 3qcent-13.1, 7p14-pter, 7q11.23–q22, 12p13.3, 13q33, 14q24.2–q32.1, 17p	3q28, 4q21.2–q24, 5p, 13q12.1–q12.2, 18p11.31-pter	13	1p32.1, 1q21.1–q21.3, 11qcent-q13.3, 19p13.3-pter, 19qcent-q13.1, 22q13.2	14
21	1q31, 3q21–q27, 5p, 8q22.1–q24.1, 11q12–q14.2, 12p	7q21.2–q21.3	7	14q, 16p13.3, 16q	9
22	1q25–32.1, 3q, 7p15.2-pter, 8q23–q24.2, 9q33–34.2, 12p, 16q23	1q21.3–22, 7q, 11qcent-q13.2, 17q23, 18p11.31-pter, 19p, 19qcent-q13.2, 20q13.1	15	1p36.3-pter, 10p15-pter, 11p15.5-pter, 16p13.3, 19q13.4-qter, 21q22.2, 22q13.2	14
23	1qcen-q31, 5p, 8q22.2–q24.2, 12p, 12qcent-q21.3, 14q32.2, 17q21.2–q24, 19p13.3-pter	1p32.1–p32.3, 3q35–q36, 9q34.2	11	2pcen-p16, 2q21.2–q31, 3p11–p12, 3q21–q28, 6pcen-p12, 6q, 7q21.1, 7q31.1–q35, 9p23-pter, 10p15, 15q21.1–q25, 18p, 22q13.2–q13.3	16

PT=primary tumour; LNM=lymph node metastasis.

and 4

**Table 4:** Chromosomal locations of DNA loss in individual patients

**ID no.**	**Common deletions**	**PT, unique deletions**	**Total no. deletions**	**LNM, unique deletions**	**Total no. deletions**
1	3p, 4p, 5q, 13q12–13	1p34-pter, 2q37-qter, 10p, 11q14-qter, 17p	9	1p2, 2q24–32, 4qcent-q28, 6qcen-q23, 9p, 14q21–23, 18q, 21	12
2	4q26-qter, 5q14–23, 13q2-qter	2q37, 3p, 6q26-qter, 8p, 10q25-qter, 11q21-qter, 18q22-qter, 21	11		3
3	1p33-pter, 2p23-pter, 3p, 4p14–15, 4q26-qter, 5q13, 6p22-pter, 8p, 9pter-q22.1, 10, 11q23-qter, 13q12–13, 13q22-qter, 17p, 18q, 19q		16	9q22.3, 16q	18
4	8p21-pter, 10q26	3p24-pter, 11q24-qter	4		2
5	3p, 11q23-qter		2	2q37, 4p15.3-qter, 5q34-qter, 7q31.3-qter, 12q22-qter	7
6	1p32–pter, 9q34, 17p	3p2-pter, 4p15.3-pter, 5q34-qter, 10, 11p15-pter, 14q31-qter, 16q, 21	11	12q24-qter, 16p, 17q24-qter, 19, 22	6
7	1p34-pter	2p25, 2q37, 3p23-pter, 4p15-pter, 5p15.1-pter, 5q35, 8q24.3-qter, 10q25-qter, 11p14, 11q23-qter, 18q22-qter	12	9q34, 17, 19, 20q13-qter, 22	6
8	3p, 5q31-qter, 7q32-qter, 8p	2q37, 6p23-pter, 11p14-pter, 18q22-qter, 21	9	4p16	5
9	2q37.2-pter, 4p16-pter, 5q35.1-qter, 12q24.31-qter, 17q25-qter	1p36-pter, 10p13-pter, 14q32.1-qter, 19q13.4-qter	9	7q36-qter, 10q26-qter	7
10	1p35-pter, 9q32-qter, 17q23-qter, 19p, 22q	5q34-qter, 16p, 17p, 19q	9	4p15.3-pter, 12q24.2-qter, 21q	8
11		5q35, 6p24-pter, 11p15, 18q22-qter, 21	5	1p35-pter, 9q34, 17p13	3
12	3p, 13q, 14q22-qter	8p22-pter, 11q14-qter, 16p12-cen	6	2p32, 4qcen-q28, 5q13, 5q21–23, 6q12–q22, 9p23	9
13	5q33-qter, 8p, 11q22-qter, 19p, 22	3p14–p25, 7q31.3-qter, 18q12-qter,19q, 21	10	1p33–35, 4p16, 6q25-qter, 16p, 16q22-qter, 17p, 20q12–13.1	12
14	1p31.1–31.2, 3p, 6q24-qter, 9p, 9q32-qter, 11p, 13q14, 14q21-qter, 15q, 16	8p, 11q21–23, 21	13	4q33-qter, 10	12
15		6q26	1	7q36	1
16	1p32-pter, 2q35-qter, 4p15.3-pter, 8p, 9p23-pter, 9q34-qter, 10q25-qter, 11p15-pter, 13q31-qter, 16p12-pter, 17p, 17q24-qter, 18q21-qter, 19p, 21, 22	3p25-pter, 5q34-qter, 10p14, 11q23-qter, 16q22-qter	21	4q33-qter, 5p15.1-pter, 7p21-pter	19
17	3p, 5q13, 10, 11q23, 13	5q34-qter, 7q32-qter, 12q24-qter, 16p, 16q22-qter, 21	11	1p36-pter, 4p15, 17q24-qter, 20p, 22	10
18	1p31.1-pter, 2q36-pter, 4q, 8p21–22, 11p15-pter, 17q, 21q22.1–22.2	2p25.1-pter, 5q33.2-qter, 7q33-qter, 10p, 12q24.2	13	4p15.2-pter, 5q13.1–13.3, 6q25.1–26, 14q23-qter, 16q22–23, 19p13.1-pter	14
19	1p13.2-pter, 2q35–37, 3p13–21.1, 3p21.3-pter, 4p13-pter, 4q28–4q34, 5q12-qter, 8p12-pter, 13q22–33, 20q12–q13.1	1q23,1q42, 2q21, 2q35–37, 6p22.3–p24, 18q11.2–q22, 20p11.2–p12, 21q11.2–q21	18	4q22–q23, 6q16.3–q21, 7q11.23–q21.1, 9p23-pter, 10q, 11q22.3–q24, 15q13–q26, 19q13.1–q13.2	18
20	6p22.3-pter, 10qcent-q21.2	2q34	3	3p, 5q, 11q22.3–q24, 13q14.1-pter	6
21	1p13.3–31.1, 3p13-pter, 4p15.1–15.2, 6q21–26, 13q, 18q12.2–22, 21qcent-q22.2		7	9p12, 9p21–23, 15q, 19p13.1-pter	11
22	4q31–q34, 15q12–q14	1p22.1–p31.1, 4p12–p15.2, 13q21.1	5	16p11.2–p14	3
23	3p13-pter, 4p15.1-pter, 4q31.2–q34, 7p	9p12	5	1p36.2, 2q33–q37.2, 5q23.1–q34, 8q13, 9q21.1–q33, 10qcen-q25.3, 11p12-pter, 11qcen-13.3, 11q22.1–q22.3, 13qcent-q13, 13q31–q33, 14q22–q23, 16q12.1–q23, 17p11.2-pter, 18q12.2–q22, 20p11.2–p12, 20q11.2–q13.2	20

PT=primary tumour; LNM=lymph node metastasis.

. Figures 1

**Figure 1:**
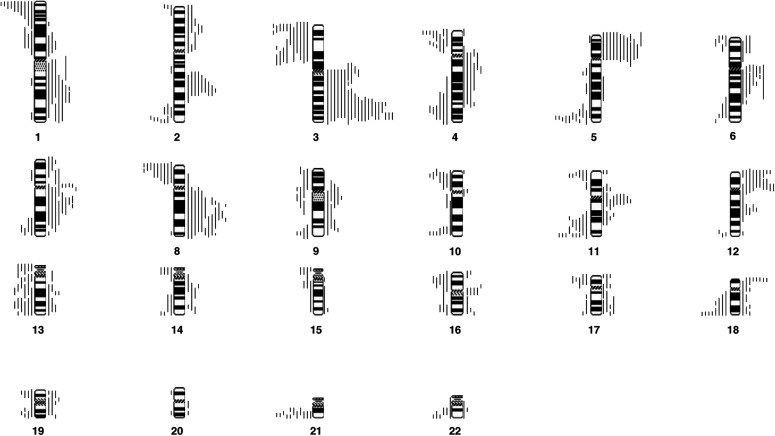
Ideogram to show the location of all the deletions and gains found in all 23 primary tumours. Lines drawn to the left of the ideogram represent the location of regions of deletion or loss. Lines drawn to the right represent the location of gains or amplifications of chromosomal material.

and 2

**Figure 2:**
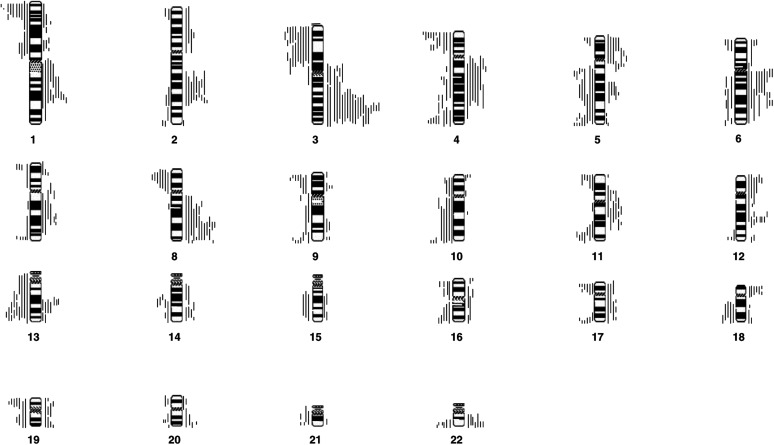
Ideogram to show the location of all the deletions and gains found in all 23 metastatic lymph node samples. Lines drawn to the left of the ideogram represent the location of regions of deletion or loss. Lines drawn to the right represent the location of gains or amplifications of chromosomal material.

depict the location of all of the chromosomal gains and losses found in our patients.

The clonal relationships (CR) between primaries and LNMs were evaluated according to a probabilistic model. These results showed a strong CR, >95% concordance in all but one case.

## DISCUSSION

CGH screens the entire genome for DNA copy number alterations, and thus yields vast quantities of information. Analysis of this information from a matched primary tumour and lymph node provides a clear picture of the presence or absence of a CR. In our study, a strong CR was found in all but one case, strongly suggesting that the cells, which were found in the lymph nodes, originate from the dominant cell population in their corresponding primary tumours.

Our cohort of patients was similar to Bockmühl's series, that is, all samples were HNSCC taken from patients with no history of previous malignancy or treatment such as radiotherapy. The cohort of 23 patients reported here is the second largest group to be studied: Bockmühl's series contained 34 patients. Kujawski's cohort (19 patients) matched the same patient criteria but limited the site to larynx. Comparison of our findings show that the frequency of 3q25–q27, 5p, 11q13–q14.1 and 1q31 gains are the same as reported previously (Bockmuhl *et al*, 1998; Bergamo *et al*, 2000; Gollin, 2001). Our results were also very similar to Kujawski *et al* (1999) with respect to gains at 3q22–q24, 8q24 and deletions at 3p22–p25, 4p15.3-pter, 4q33-qter, 7q22 and 18q23. Bockmühl's series (2000) revealed a higher incidence of all these aberrations, which could be explained by different and/or more sensitive CGH analysis software. Interestingly, in our and Bockmühl's cohort, aberrations were found that were absent in Kujawski's series, for example, deletions at 1p34.2-pter (found in 43.5% of the PT and 39.1% of the LNM in our cohort, and 65 and 27%, respectively, in Bockmühl's cohort) and 19p13.3-pter (found in 17.4% of PT samples and 30.4% of LNM in our cohort, and 15 and 63%, respectively, in Bockmühl's cohort). The significance of these differences is unclear, but they seem to highlight the need for analysis of specific subsites of HNSCC to clarify whether these genetic variations reflect different anatomical locations and if so their potential as diagnostic markers. This study, although reporting one of the largest HNSCC cohorts, becomes relatively small if subdivided by anatomical site or staging; therefore, such analysis was not undertaken.

The similar number of unique random aberrations in both the primary and LNM samples could be explained by continued clonal evolution after the divergence of the metastatic cells. If this is the case, the unique changes in the primary tumour are most likely to be incidental to the metastatic progression. Therefore, the common aberrations found in both paired samples as well as the unique changes in the LNM probably underlie the tumour cell's ability to metastasise and proliferate.

Our data show associations between gains of chromosomal material on 6q and 22q in the LNM and not in the primary tumour samples. Detailed analysis of the location of the DNA gain on 6q show two frequent areas –6q13 and 6q22.3, both more frequent in the LNM than PT, but not of statistical significance (*P*=0.2 and 0.11, respectively). There were also more LNM samples with gains of DNA material on 22q. Bockmühl's cohort (along with Welkoborsky *et al*, 2000) found equal numbers of 22q aberrations between PT and LNM, whereas Kujawski had very few in either sample (Table 2). Although the difference in the expression of this gain on 22q was not statistically significant in our cohort (McNemar's test *P*=0.07, Fisher's exact test *P*=0.056), other studies have found a loss of heterozygosity at this site (Poli-Frederico *et al*, 2000; dos Reis *et al*, 2002) and an association with poor prognosis (Ashman *et al*, 2003). Two genes are located on 22q in close proximity to each other: STMY3 at 22q11.2 and the BCR gene. The STMY3 gene encodes for stromelysin III, a member of the matrix metalloproteinase family that is involved in the physiological and pathological control of extracellular matrix remodelling. Stromelysin III has been found to be overexpressed in the stromal cells of invasive breast carcinoma (Nakopoulou *et al*, 2002). Whether this chromosomal gain on 22q only occurred in a few cells in the PT, and therefore, was not detectable by CGH, but gave this subclone a competitive advantage to metastasise or whether this expression occurred after migration is not clear from this study. Future work focusing on such genes in the PT will hopefully clarify their importance in the development of metastasis.

The CGH results from our work and the previous two major studies discussed here have upheld the clonality of the metastatic cells; but no obvious single or group of aberrations appears to cause metastatic spread in HNSCC, that is, a distinct, common tumour progression pathway is not evident. It must be noted that 18 of 23 of our cohort had advanced T3 or T4 carcinomas, and thus more incidental aberrations may be present and obscure the genotype for metastatic progression. A similar preponderance towards more advanced tumours is true for Bockmuhl's cohort.

Another theory is that some primaries are ‘preconfigured to metastasise’ by the time of diagnosis (Ramaswamy *et al*, 2003). Both Welkoborsky *et al* (2000) and Bockmuhl *et al* (2002) attempted to address the differences between nonmetastasising and metastasising HNSCC in their studies; however, disparate results have been reported. Welkoborsky and co-workers reported over-representation of DNA on 1p and 7p exclusively in node-negative PT and over-representation of DNA on 1q, 11q and 22q were in node-positive PT. Bockmuhl *et al* (1997) analysed 29 metastasising and 19 nonmetastasising HNSCC, and showed a preferential association between gain of DNA on 5p, 6p and 7p and node-negative PT. Node-positive tumours were characterised by deletions on chromosomes 7q, 10q 11p, 11q 15q and 20p, as well as gains on chromosomes 19q and 20q. The more recent, larger, study by Bockmuhl *et al* (2002) that included some of the patients analysed previously also found an association between deletions of 10q, 11p and 11qter and node-positive PT. Whether these differences are simply due to the relatively small number of tumours studied or truly reflect the complex genetic make-up of HNSCC remain to be elucidated.

Although the technique of CGH has limitations, that is, balanced translocations, rearrangements and/or ploidy change cannot be recognised, and no obvious genetic metastatic signature could be identified; the CGH data from all three studies do confirm the clonal relationship of the primary and LNM. Furthermore, CGH remains a useful tool to guide the application of higher-resolution techniques such as loss of heterozygosity studies and mutational analysis, ultimately speeding up the identification of the critical genes involved in HNSCC tumorigenesis. In the future, once such genes have been categorised, new technologies like DNA microarrays, which simultaneously analyse the expression of vast repertoires of genes in individual tumours, will be used to predict the clinical characteristics of a patient's tumour at the time of diagnosis allowing the optimisation of the treatment regimen.
