# Can a genetic signature for metastatic head and neck squamous cell carcinoma be characterised by comparative genomic hybridisation?

**DOI:** 10.1038/sj.bjc.6602114

**Published:** 2004-08-24

**Authors:** H S Patmore, N E Ashman James, L Cawkwell, A MacDonald, N D Stafford, J Greenman

**Correction to:** *British Journal of Cancer* (2004) **90**, 1976–1982. doi:10.1038/sj.bjc.6601756

Owing to an author error, the second author's name in the above paper was printed incorrectly. The correct author listing is:

HS Patmore, JNE Ashman, L Cawkwell, A MacDonald, ND Stafford and J Greenman

